# ABDGAN: Arbitrary Time Blur Decomposition Using Critic-Guided TripleGAN

**DOI:** 10.3390/s24154801

**Published:** 2024-07-24

**Authors:** Tae Bok Lee, Yong Seok Heo

**Affiliations:** 1 Department of Artificial Intelligence, Ajou University, Suwon 16499, Republic of Korea; dolphin0104@ajou.ac.kr; 2 Department of Electrical and Computer Engineering, Ajou University, Suwon 16499, Republic of Korea

**Keywords:** single image deblurring, arbitrary time blur decomposition, continuous motion deblurring, Triple Generative Adversarial Networks, critic-guided loss, pairwise order-consistency loss

## Abstract

Recent studies have proposed methods for extracting latent sharp frames from a single blurred image. However, these methods still suffer from limitations in restoring satisfactory images. In addition, most existing methods are limited to decomposing a blurred image into sharp frames with a fixed frame rate. To address these problems, we present an Arbitrary Time Blur Decomposition Triple Generative Adversarial Network (**ABDGAN**) that restores sharp frames with flexible frame rates. Our framework plays a min–max game consisting of a generator, a discriminator, and a time-code predictor. The generator serves as a time-conditional deblurring network, while the discriminator and the label predictor provide feedback to the generator on producing realistic and sharp image depending on given time code. To provide adequate feedback for the generator, we propose a critic-guided (CG) loss by collaboration of the discriminator and time-code predictor. We also propose a pairwise order-consistency (POC) loss to ensure that each pixel in a predicted image consistently corresponds to the same ground-truth frame. Extensive experiments show that our method outperforms previously reported methods in both qualitative and quantitative evaluations. Compared to the best competitor, the proposed ABDGAN improves PSNR, SSIM, and LPIPS on the GoPro test set by 16.67%, 9.16%, and 36.61%, respectively. For the B-Aist++ test set, our method shows improvements of 6.99%, 2.38%, and 17.05% in PSNR, SSIM, and LPIPS, respectively, compared to the best competitive method.

## 1. Introduction

Single image deblurring is one of the most classic yet challenging research topics in the field of image restoration, which aims to restore a latent sharp image from a blurred image. Recently, deep-learning-based methods have achieved remarkable success in image deblurring by training models on large-scale synthetic deblurring datasets (e.g., GoPro [1], DVD [2] and REDS [3]). These datasets have been suggested to synthesize blurred images by averaging consecutive sharp frames sampled from videos. They are based on the premise that the motion blur can be seen as the accumulation of movements that occurred during the camera exposure duration [1]. Motivated by this, many methods have made significant progress in estimating a sequence of sharp frames from an observed blurred image, which is also known as the blur decomposition task [4].

Due to the complex and ill-posed nature of blur decomposition [5], existing methods [4,5,6] face significant challenges. There is substantial room for improvement in generating visually pleasing, high-quality frames. In addition, most of them have been designed to restore a fixed number of frames using supervised learning. This limits the flexibility and applicability of these models, as adjusting the network architecture or training procedure is necessary to produce different numbers of frames. One of the practical approaches for restoring a flexible number of frames involves first extracting a fixed number of sharp frames using blur decomposition methods. Subsequently, video interpolation is applied to these frames. However, this approach may not be optimal, since inaccurate blur decomposition can lead to degraded quality of the interpolated frames.

In this work, we propose an Arbitrary Time Blur Decomposition Using Critic-Guided Triple Generative Adversarial Network (ABDGAN) (This article is based on Chapter 5 of the first author’s Ph.D. thesis [7]). This approach restores a sharp frame with an arbitrary time index from a single blurred image, a task we refer to as arbitrary time blur decomposition. One of the main challenges for this problem is the lack of ground-truth (GT) images for every continuous time within exposure duration in existing synthetic datasets [1]. Recent synthetic deblurring datasets (e.g., GoPro [1]) have used a range of {7,9,11,13} sharp frames to synthesize a blurred image. This means that there are only a limited number of timestamps for each blurred image, with no GT images for all continuous time codes over the exposure time. In this circumstance, when the models are trained by supervised fashion using these datasets, they may not be able to effectively restore images at timestamps that are not present in the training set [8].

As a departure from previous blur decomposition methods that rely on supervised learning, we propose a semisupervised framework. To this end, we adopt the TripleGANs framework [9] which consists of three players, including a generator, a discriminator, and a classifier. For the blur decomposition task, we modify the role of three players and the objective functions for the generator and the label predictor. Specifically, our ABDGAN plays a min–max game of three players consisting of a time-conditional deblurring network *G*, a discriminator *D*, and a label predictor *C*. Our *G* takes a pair of a time code and a blurred image as inputs, and restores a corresponding sharp moment occurring within exposure duration. Meanwhile, *D* estimates the probability of whether given images are real or fake. Concurrently, *C* predicts the time code when the blurred image and latent sharp image are jointly given. Since the training (real) data do not include sharp frames for every consecutive time, *C* is trained not only on real images but also on the generated sharp images by *G*. However, in our framework, a naïve adoption of TripleGAN [9]’s approach often faces unstable training of *C* due to the distribution discrepancy problem [10] between real and fake images. This arises, especially in the early training phase, where the restored images from *G* may not match the real data distribution well enough. This makes it difficult for *C* to correctly predict the time codes for both real and fake images.

To mitigate this, unlike the original TripleGANs [9], which directly utilizes fake samples obtained by the generator for training the classifier, our *D* assists *C* in filtering out unrealistic fake samples. To this end, we propose a critic-guided (CG) loss, allowing our *C* to train using reliable fake images generated from *G* based on the feedback from the critic *D*.

On the other hand, it is a highly ill-posed problem to recover a temporal ordering of frames from a single blurred image, because motion blur is caused by averaging that ruins the temporal ordering of the instant frames [5]. To address the challenge of frame-order recovery, most existing methods [5,6,11,12] utilize the pairwise ordering-invariant (POI) loss [5]. The POI loss is invariant to the order by utilizing the average of two temporal symmetric frames and the absolute value of their difference. This approach enables the network to choose which frame among symmetric frames to generate during training [5]. As a result, this loss function effectively facilitates stable network convergence by preventing temporal shuffling and ensuring temporal consistency among predicted frames. However, it is suboptimal because pixel-level consistency is not guaranteed, potentially resulting in each pixel in a predicted image corresponding to different GT frames.

To address this problem, we propose a pairwise order-consistency (POC) loss that alleviates the problem of pixel-level inconsistency inherent in the existing POI loss [5]. Our POC loss shares similarities with the POI loss by including temporal symmetric frames in the loss function. However, our POC loss differs from the POI loss in that the POI loss implicitly matches pairs of estimated frames and GT frames to define the loss, while our POC loss explicitly determines these pairs. Specifically, the proposed POC loss starts by determining whether the temporal order of predicted sharp images aligns with the GT order or its reverse. This preliminary step enables us to determine which specific GT image and predicted image should be optimally minimized. Following this, we ensure that each pixel in a predicted image consistently matches the corresponding pixel in the same ground-truth frame by rigorously enforcing across all pixels.

**Figure 1:**
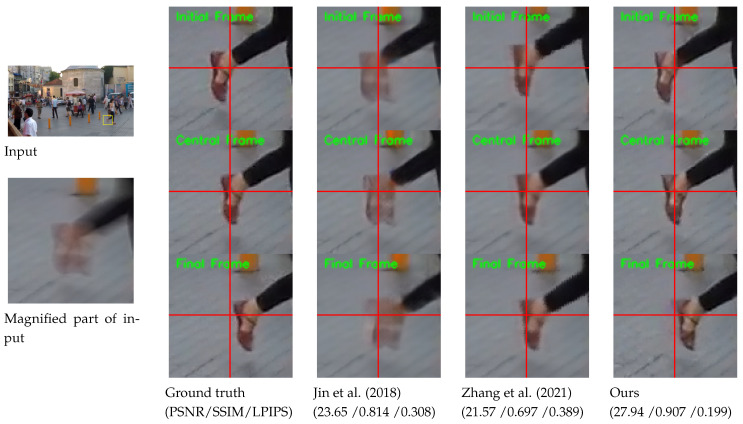
Example of blur decomposition results on GoPro test set [1]. For clarity, we display the magnified parts of the output images. The figure exemplifies the superiority of our model compared with previous methods [5,13]. Horizontal and vertical lines are marked at the center coordinate of each image to provide clear observation of object movements between consecutive frames. The first, second, and third rows of output images indicate the initial frame, central frame, and final frame among the predicted video sequence from an input image. For quantitative comparison, we calculate the PSNR, SSIM, and LPIPS values by averaging those of the initial, central, and final frames.

Figure 1 exemplifies the superiority of our model compared with previous methods [5,13]. Unlike existing methods, our model can restore highly accurate dynamic motion from a blurred image. Moreover, our model can extract sharp sequences at any desired frame rate, while competing methods are constrained to restoring a predetermined number of frames.

Our main contributions can be summarized as follows.

- We propose Arbitrary Time Blur Decomposition Using Critic-Guided TripleGAN (ABDGAN), a semisupervised learning approach, to extract an arbitrary sharp moment as a function of a blurred image and a continuous time code.
- We introduce a critic-guided (CG) loss, which addresses the issue of training instability, especially in the early stages, by guiding the label predictor to learn from trustworthy fake images with the assistance of the discriminator.
- We introduce a pairwise order-consistency (POC) loss, designed to guarantee that every pixel in a predicted image consistently matches the corresponding pixel in a specific ground-truth frame.
- Our extensive experiments demonstrate that our method surpasses existing methods in restoring high-quality frames at the GT frame rates, and consistently produces superior visual quality at arbitrary time codes.

The remainder of this paper is organized as follows. In Section 2, we review previous works on image deblurring. Section 3 presents the details of our proposed ABDGAN. Section 4 analyzes the experimental results of the proposed method. Finally, in Section 6, we discuss the conclusions and future works.

## 2. Related Works

In this section, we provide an overview of single image deblurring and blur decomposition methods utilizing deep learning. In Table 1, we briefly categorize the deblurring methods based on their ability to recover a single middle frame, a fixed number of multiple frames, and an arbitrary number of multiple frames. We also introduce the TripleGAN, which is closely related to our proposed approach.

### 2.1. Image Deblurring

In general, image deblurring refers to restoration of a sharp image from an observed blurred image [28]. Recently, numerous studies in this field have achieved remarkable success based on deep learning. Early methods [14,15,16] utilized deep learning to estimate the blur kernel. On the other hand, Nah et al. [1] proposed to directly restore the sharp image without additional blur kernel estimation step, and developed a multiscale deblurring network that performs restoration in a coarse-to-fine manner. Motivated by the success of [1], numerous methods, such as multiscale recurrent model [17], multipatch hierarchical network [18], and multi-input–multi-output unet [19] have been proposed and have achieved promising results. On the other hand, generative adversarial networks (GANs) have been widely used in image deblurring. Kupyn et al. [20] proposed DeblurGAN, which adopts conditional GAN for motion deblurring. Extending this, DeblurGAN-v2 [21] is proposed to use the relativistic discriminator and feature pyramid deblurring network for motion blur. DBGAN [22] utilizes GANs to learn image deblurring and blurring process. Furthermore, the GAN-based method is also extended to video deblurring methods, such as DBLRGAN [23]. In contrast, our approach diverges from these methods by adopting TripleGANs [9] to the blur decomposition task, which comprises three core networks for adversarial learning. Recently, Kong et al. [24] presented a frequency-domain-based self-attention solver and a discriminative frequency-domain-based feedforward network to enhance the deblurring performance. Roheda et al. [25] proposed a network architecture based on the higher-order Volterra filters for image and video restoration. Mao et al. [26] proposed an adaptive patch exiting reversible decoder that maximizes image deblurring performance while maintaining memory efficiency.

### 2.2. Blur Decomposition

The blur decomposition [4] is used to extract a sharp sequence from a blurred image. As a pioneer, Jin et al. [5] proposed the generation of sharp image sequences by cascading multiple deblurring networks. Instead of using multiple networks, Purohit et al. [11] proposed a two-stage framework based on recurrent networks. Unlike [5,11], which require multiple training stages, Argaw et al. [6] developed an end-to-end trainable framework. To obtain a large number of output frames, Zhang et al. [12] proposed a cascaded structure with three GANs, and each generator is trained to extract seven consecutive frames from an input image. Zhang et al. [13] proposed a motion-offset estimation-based model, in which they train the motion offset generation module first, and then attach it to the deblurring network. Zhong et al. [4] tackled the motion ambiguity problem in blur decomposition by directly conditioning the motion guidance. Despite these efforts, most existing methods require changing the network architecture or retraining according to changes of the number of frames. Recently, ref. [27] mitigated this shortcoming by using a control factor for face image deblurring. However, this method can only be applied to face images, which limits the generalization ability to process large and complex motion in natural scenes. In this work, our goal is to recover the sharp moment from a given blurred image at arbitrary and continuous time codes. This is achieved without the need for retraining or changing the network architecture.

### 2.3. Triple Generative Adversarial Networks

GANs [29] have gained significant attention in image synthesis. Among the various extensions of GANs, conditional GANs [30] were developed to perform conditional image synthesis. However, most of these methods rely on supervised learning [9], which requires fully labeled images in the dataset. To learn conditional image synthesis with partially labeled data, TripleGAN [9] employs an additional label predictor as a pseudo-label generator and plays a min–max game of three networks. To address the distribution discrepancy issue, ref. [10] ensembled multiple classification networks and utilized the feature matching loss between the generated and real samples. Inspired by previous works, we extend the application scopes of TripleGANs [9,10] to the blur decomposition task. However, the simple application of TripleGAN’s method leads to unstable network training due to the two main issues: (1) lack of real images for continuous time codes, and (2) distribution discrepancy between real images and fake images. Our ABDGAN framework is mainly designed to effectively resolve the above problems.

## 3. Proposed Method

Let $b\in R^{H\times W\times 3}$ and t∈[0,1] represent a single blurred image and a temporal index within the normalized exposure time, respectively. Our goal is to restore a specific sharp moment ${\hat{s}}_{t}\in R^{H\times W\times 3}$ conditioned on *b* and *t*. One of the major challenges in our goal is to predict the sharp moment ${\hat{s}}_{t}$ for any continuous time code, particularly when the training data contain very few ground-truth images for continuous time code. To overcome this, the proposed ABDGAN utilizes semisupervised learning by leveraging both labeled and unlabeled data. Here, the labeled dataset $\left\{b,t,s_{t}\right\}$ is sampled from the real-data distribution $p_{d}$. It explicitly contains ground-truth sharp images $s_{t}$ corresponding to each *b* and *t*. In contrast, the unlabeled data, the set $\left\{b,t\right\}∼p_{d}$, indicate that there is no ground-truth sharp image $s_{t}$. By leveraging both labeled and unlabeled data, the proposed method aims to predict an accurate sharp moment for any continuous time code. This is achieved despite the scarcity of ground-truth sharp images in the training dataset.

**Figure 2:**
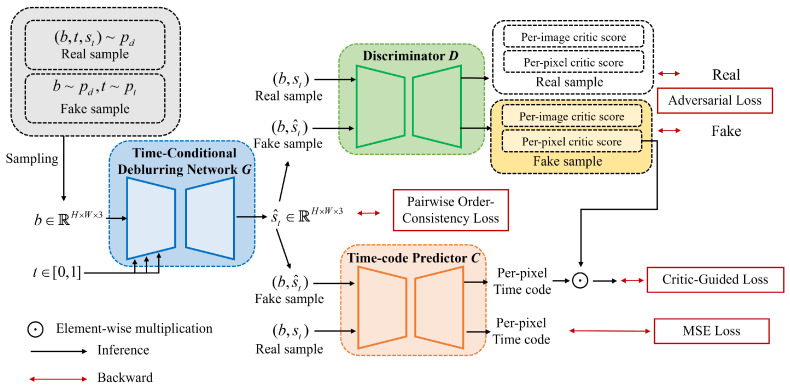
The pipeline of ABDGAN. During training, ABDGAN plays a min–max game of the three networks *G*, *D*, and *C*. For every iteration, they are optimized alternatively with the proposed critic-guided loss. For testing, *G* is only used to render the sharp image at arbitrary t∈[0,1] from a blurred image.

**Learning Arbitrary Time Blur Decomposition based on TripleGANs.** Inspired by TripleGANs [9,10], which achieved successful results in conditional image synthesis in a semisupervised manner, our proposed ABDGAN introduces a new strategy. It plays a min–max game consisting of three players: a time-conditional deblurring network *G*, a discriminator *D*, and a time-code predictor *C*, as depicted in Figure 2. As mentioned earlier, one of our major goals is to train *G* to predict any sharp moment corresponding to arbitrary time code and a blurred image. To achieve this, our *D* plays a role of providing adversarial feedback for *G* to restore realistic sharp images. Simultaneously, the main role of *C* is to provide precise feedback so that *G* generates an accurate temporal sharp moment corresponding to the input time code among latent sharp motions within the blurred image.

Concretely, given a pair of *b* and t∈[0,1], the proposed time-conditional deblurring network *G* outputs a sharp frame ${\hat{s}}_{t}$, which is written as ${\hat{s}}_{t}=G\left(b,t\right)$. As illustrated in Figure 2, *D* receives a pair (b,s) as input, where *s* represents either a real sharp image $s_{t}$ or restored sharp image ${\hat{s}}_{t}$ from *G*. During training, *D* is trained to predict whether the input comes from the real-data distribution $p_{d}\left(b,s\right)$ or the fake-data distribution $p_{g}\left(b,s\right)$. Structurally, we exploit a UNet discriminator [31] for our *D*’s architecture. This architecture involves an encoder that outputs a per-image critic score $D_{e}\left(\cdot \right)$ and a decoder that outputs a per-pixel critic score $D_{d}\left(\cdot \right)$. Given a pair (b,s) as input, where *s* represents either a real sharp image $s_{t}$ or restored sharp image ${\hat{s}}_{t}$, our *C* is trained to accurately predict the corresponding temporal code, as depicted in Figure 2. Since our *C* is trained using fake images as well as real images, *C* can provide adequate feedback to our *G* to ensure that the restored sharp moment aligns accurately for arbitrary time code. Considering that image restoration is a pixel-by-pixel dense prediction task [32,33,34], we employ a UNet-based architecture [35] for our *C* to provide per-pixel feedback on *t* for *G*. Let the temporal code map $t_{m}\in R^{H\times W}$ denote a 2-dimensional matrix filled with *t* as $t_{m(i,j)}=t$ for every pixel coordinate (i,j). Given an input pair of $\left(b,s_{t}\right)$, our *C* fuses *b* and $s_{t}$ using channel-wise concatenation, and outputs pixel-wise time-code map ${\hat{t}}_{m}\in R^{H\times W\times 1}$, which is written as ${\hat{t}}_{m}=C\left(b,s_{t}\right)$.

**Pairwise-order consistency (POC) loss.** For training our *G* with the labeled data $\left\{b,t,s_{t}\right\}∼p_{d}$ more effectively, we propose our POC loss. Unlike conventional POI loss [5] employed in previous studies [5,6,11,12], our proposed POC loss offers distinct advantages by enforcing stronger constraints on the temporal order of predicted frames. The proposed POC loss results in significant improvements in the accuracy and visual quality of predicted frames compared to existing POI loss.

**Critic-guided (CG) loss.** As mentioned earlier, the distribution discrepancy problem is one of the crucial challenges in training TripleGAN-based framework [10]. The limited number of labeled data may not be sufficient for our *G* to effectively learn to restore a sharp moment when the input temporal code is absent in the training data. To overcome this, our *C* is trained not only with labeled sharp images but also with fake sharp images restored by *G* with unlabeled data. However, especially in the early training phase, a distribution discrepancy can arise between real and fake images. This poses a challenge for our *C*, which is trained to predict correct time codes for both real and fake images. To address this, we propose our CG loss, optimizing *C* using realistic fake images by leveraging the decision made by *D*.

**Table of notation.** To ensure clarity and consistency, Table 2 shows a concise summary of the notations used throughout this paper. Unless stated otherwise, we maintain consistency in notation.

In the following, we provide explanations of our pairwise-order consistency (POC) loss in Section 3.1, and the critic-guided (CG) loss in Section 3.2. The entire training procedure is described in Section 3.3.

### 3.1. Pairwise-Order Consistency Loss

In Appendix A, we describe the limitations of the existing POI loss [5]. Based on this analysis, we introduce our POC loss, which is designed to overcome the shortcomings. Let $\left\{b,t,\bar{t},s_{t},s_{\bar{t}}\right\}∼p_{d}$ denote the sampled set from the dataset, where $\bar{t}=1-t$. That is, $\left(s_{t},s_{\bar{t}}\right)$ is a pair of GT symmetric frames for the central frame $s_{t=0.5}$. This implies that $\left(s_{t},s_{\bar{t}}\right)$ is a pair of GT symmetric frames for the central frame $s_{t=0.5}$. Then, we can obtain ${\hat{s}}_{t}=G\left(b,t\right)$ and ${\hat{s}}_{\bar{t}}=G\left(b,\bar{t}\right)$. Without loss of generality, *t* and $\bar{t}$ satisfy $t<\bar{t}$. Then, the proposed POC loss $L_{G}^{\mathrm{POC}}$ is defined as follows:

(1) $$\begin{array}{cc} L_{G}^{\mathrm{POC}}=\; & \left\{\begin{array}{c} ∥\hat{s_{t}}-s_{t}{∥}_{1}+{∥{\hat{s}}_{\bar{t}}-s_{\bar{t}}∥}_{1}\;\mathrm{if}\;\Phi \left(s_{t},{\hat{s}}_{t}\right)<\Phi \left(s_{t},{\hat{s}}_{\bar{t}}\right)\\ ∥\hat{s_{t}}-s_{\bar{t}}{∥}_{1}+{∥{\hat{s}}_{\bar{t}}-s_{t}∥}_{1}\;\mathrm{otherwise} \end{array}\right. \end{array},$$

where Φ(·,·) is the $L_{1}$ distance metric between two images. The proposed $L_{G}^{\mathrm{POC}}$ utilizes Φ(·,·) to assess whether the temporal order of predicted sharp images aligns with the GT order or its reverse. If $\Phi \left(s_{t},{\hat{s}}_{t}\right)<\Phi \left(s_{t},{\hat{s}}_{\bar{t}}\right)$, this indicates that the GT frame $s_{t}$ is closer to its corresponding predicted frame ${\hat{s}}_{t}$ than to the opposite time-symmetric predicted frame ${\hat{s}}_{\bar{t}}$. Consequently, this suggests that ${\hat{s}}_{t}$ and ${\hat{s}}_{\bar{t}}$ are correctly aligned with GT temporal order of sharp frames $s_{t}$ and $s_{\bar{t}}$, respectively. Based on this, we directly minimize the sum of individual $L_{1}$ distance between each predicted frame and its correct GT frame. Conversely, if $\Phi \left(s_{t},{\hat{s}}_{t}\right)\geq \Phi \left(s_{t},{\hat{s}}_{\bar{t}}\right)$, it implies that the predicted frames ${\hat{s}}_{t}$ and ${\hat{s}}_{\bar{t}}$ are aligned with the reverse order of GT frames. In such a case, we minimize the sum of the $L_{1}$ distance between ${\hat{s}}_{t}$ and $s_{\bar{t}}$, and between ${\hat{s}}_{\bar{t}}$ and $s_{t}$. Our POC loss marks a departure from the existing POI loss [5] (Equation (A1)). It introduces stricter constraints to ensure that every pixel in the predicted image aligns consistently with the same ground-truth (GT) frame. This enhancement substantially improves the accuracy and reliability of frame prediction.

### 3.2. Critic-Guided Loss

The proposed CG loss trains *C* with trustworthy fake samples induced by the pixel-wise critic score $D_{d}\left(\cdot \right)$, which represents the pixel-wise probability map predicted by *D*. The *D* is trained to predict a probability value close to 1 when the input sample is as realistic as the real image, and 0 for vice versa [29]. If the output probability value of *D* for a fake image generated by *G* is 0.5, this means that *G* generates a sharp image whereby *D* cannot distinguish between real and fake [29]. Based on this, we consider the case where the output of *D* is greater than 0.5 for fake data to be trustworthy (realistic) fake data. Accordingly, the sigmoid-based soft threshold function σ(·) is pixel-wisely applied to ${\left[D_{d}\left(\cdot \right)\right]}_{\left(i,j\right)}$, as $\sigma \left(x\right)=\frac{1}{1+e^{-k(x-x_{0})}}$, where (i,j) indicates the pixel coordinate. Here, $x_{0}=0.5$ is the *x* value of the middle point of the sigmoid curve, and k=15 is the steepness of the sigmoid curve. From this, we can obtain the weighting mask by applying σ(·) to the outputs of $D_{d}$. For simplicity, we denote $\sigma ({\left[D_{d}\left({\hat{s}}_{t^{\prime }}\right)\right]}_{\left(i,j\right)})$ and $\sigma ({\left[D_{d}\left({\hat{s}}_{{\bar{t}}^{\prime }}\right)\right]}_{\left(i,j\right)})$ as $\sigma_{t^{\prime }\left(i,j\right)}$ and $\sigma_{{\bar{t}}^{\prime }\left(i,j\right)}$ for each pixel (i,j), respectively. Given $\left\{b,t,\bar{t},s_{t},s_{\bar{t}}\right\}∼p_{d}$ sampled from the dataset and the randomly sampled time codes $\left\{t^{\prime },{\bar{t}}^{\prime }\right\}∼p_{t}$, our CG loss $L_{C}^{\mathrm{CG}}$ is defined as follows:

(2) $$\begin{array}{ccc} \; & L_{C}^{\mathrm{CG}}=\; & \left\{\begin{array}{c} \sum_{i,j}(\sigma_{t^{\prime }\left(i,j\right)}\cdot {\left({\hat{t}}_{m(i,j)}^{\prime }-t_{m(i,j)}^{\prime }\right)}^{2}+\\ \;\;\sigma_{{\bar{t}}^{\prime }\left(i,j\right)}\cdot ({\hat{\bar{t}}}_{m(i,j)}^{\prime }-{\bar{t}}_{m(i,j)}^{\prime }{)}^{2})\;\mathrm{if}\;\Phi \left(s_{t},{\hat{s}}_{t}\right)<\Phi \left(s_{t},{\hat{s}}_{\bar{t}}\right)\\ \sum_{i,j}(\sigma_{t^{\prime }\left(i,j\right)}\cdot {\left({\hat{t}}_{m(i,j)}^{\prime }-{\bar{t}}_{m(i,j)}^{\prime }\right)}^{2}+\\ \;\;\sigma_{{\bar{t}}^{\prime }\left(i,j\right)}\cdot {\left({\hat{\bar{t}}}_{m(i,j)}^{\prime }-t_{m(i,j)}^{\prime }\right)}^{2})\;\mathrm{otherwise} \end{array}\right., \end{array}$$

where Φ(·,·) is the $L_{1}$ distance metric between two images. As a result of this collaboration with *D*, our *C* is naturally liberated from the problem of distribution discrepancy between real samples and fake samples. Since our *C* learns with the realistic fake samples generated using arbitrary value of t∈[0,1], it overcomes the problem of the limited values of *t* in the training dataset.

### 3.3. Training Objectives of ABDGAN

Similar to [9,10], the proposed ABDGAN plays a min–max game of the three networks *D*, *C*, and *G*. Algorithm 1 briefly outlines the optimization process of our ABDGAN. In Algorithm 1, *M* denotes the total number of training pairs of $\left(b,s_{t}\right)$. *f* indicates the integer frame index among the total number of sharp frames *F*. By calculating $t=\frac{f-1}{F-1}$, *t* represents the temporal code within the normalized exposure time.
**Algorithm 1** Entire training procedure of ABDGAN**Input:** Training data $X=\{\left(b^{i},s_{t}^{i}\right)|i\in \left[1,M\right]\}$, $s^{i}=\left\{s_{t}^{i}|t=\frac{f-1}{F-1},f\in \left[1,F\right]\right\}$ **Initialize:**
*D*, *C*, *G*, Adam [36] learning rate $\ell_{D},\ell_{C}$ and $\ell_{G}$, and batch size of *n*. Set balancing parameters between losses $\lambda_{POC},\lambda_{C}$ and $\lambda_{D}$. **Initialize:**
N= Number of total training iterations1:**for** iteration iter=1,2,…,N **do**2:    **Update *D*** using Algorithm 23:    **Update *C*** using Algorithm 34:    **Update *G*** using Algorithm 45:**end for**


**Algorithm 2** Training of the discriminator *D*
1:Sample a batch of $\left\{b,s_{t}\right\}∼p_{d}$ of size *n*, and a batch of $\left\{t^{\prime }\right\}∼p_{t}$ of size *n*.2:Generate fake samples using *G* as:              ${\hat{s}}_{t^{\prime }}\leftarrow G\left(b,t^{\prime }\right)$3:Compute $L_{D}$ using real samples and fake samples by Equation (3)4:Update the parameters of *D* using the gradient of $L_{D}$ by Adam [36]


**Training of the discriminator** ***D*****.** As described in Algorithm 2, the objective of *D*, $L_{D}$, is to correctly determine whether the given samples are real or fake. For this purpose, *D* is optimized to maximize the log probability of real samples and minimize the log probability of fake samples. Subsequently, $L_{D}$ consists of $L_{D_{R}}$ and $L_{D_{F}}$, which represent the losses for the real samples and fake samples, respectively. The $L_{D}$ is defined as follows:

(3) $$\begin{array}{cc} L_{D}= & -\underset{\left(b,s_{t}\right)∼p_{d}}{E}\left[\log D_{e}\left(b,s_{t}\right)+\sum_{i,j}\log{\left[D_{d}\left(b,s_{t}\right)\right]}_{\left(i,j\right)}\right]\\ \; & +\underset{b∼p_{d},t^{\prime }∼p_{t}}{E}\left[\log D_{e}\left(b,{\hat{s}}_{t^{\prime }}\right)+\sum_{i,j}\log{\left[D_{d}\left(b,{\hat{s}}_{t^{\prime }}\right)\right]}_{\left(i,j\right)}\right], \end{array}$$

where $D_{e}\left(\cdot \right)$ is the per-image critic score, measured by encoder of *D*. ${\left[D_{d}\left(\cdot \right)\right]}_{\left(i,j\right)}$ represents the per-pixel critic score measured by decoder of *D* at pixel coordinate (i,j).

**Training of the time-code predictor** ***C*****.** The details of the training scheme of *C* are described in Algorithm 3. One key aspect of our ABDGAN is that our *C* is trained to predict accurate time code for both labeled and unlabeled data. For this, the loss function for training *C*, $L_{C}$, is defined as the sum of two regression loss functions $L_{C}^{L}$ and $L_{C}^{\mathrm{CG}}$ (Equation 2). The regression loss function for labeled data, $L_{C}^{L}$, is defined using the the labeled data $\left\{b,t,\bar{t},s_{t},s_{\bar{t}}\right\}∼p_{d}$, which can be viewed as real images sampled from the training dataset. The proposed $L_{C}^{L}$ is defined as follows:

(4) $$\begin{array}{ccc} \; & L_{C}^{L}=\; & \left\{\begin{array}{c} ∥{\hat{t}}_{m}-t_{m}{∥}_{2}+{∥{\hat{\bar{t}}}_{m}-{\bar{t}}_{m}∥}_{2}\;\mathrm{if}\;\Phi \left(s_{t},{\hat{s}}_{t}\right)<\Phi \left(s_{t},{\hat{s}}_{\bar{t}}\right)\\ ∥{\hat{t}}_{m}-{\bar{t}}_{m}{∥}_{2}+{∥{\hat{\bar{t}}}_{m}-t_{m}∥}_{2}\;\mathrm{otherwise} \end{array}\right., \end{array}$$

where ${\hat{t}}_{m}$ and ${\hat{\bar{t}}}_{m}$ denote $C(b,s_{t})$ and $C(b,s_{\bar{t}})$, respectively. For unlabeled data, as described in Section 3.2, our *C* is trained using the proposed CG loss $L_{C}^{\mathrm{CG}}$ (Equation (2)). Both $L_{C}^{L}$ and $L_{C}^{\mathrm{CG}}$ are defined depending on the estimated order using *G* and the labeled data $\left\{b,t,\bar{t},s_{t},s_{\bar{t}}\right\}$. This allows *C* to predict a consistent temporal order between time codes with ground-truth images and arbitrary symmetric time codes. Overall, total loss $L_{C}$ is defined as follows:

(5) $$L_{C}=\underset{\left(b,t,\bar{t},s_{t},s_{\bar{t}}\right)∼p_{d}}{E}L_{C}^{L}+\underset{\left(b,t,\bar{t},s_{t},s_{\bar{t}}\right)∼p_{d},t^{\prime }∼p_{t}}{E}L_{C}^{\mathrm{CG}}.$$

**Algorithm 3** Training of the time-code predictor *C*1:Sample a batch of labeled data $\left\{b,t,\bar{t},s_{t},s_{\bar{t}}\right\}∼p_{d}$ of size *n*,Sample a batch of unlabeled data $\left\{t^{\prime },{\bar{t}}^{\prime }\right\}∼p_{t}\left(t^{\prime }\right)$ of size *n*2:Predict the time code matrix on labeled data by:              ${\hat{t}}_{m}\leftarrow C\left(b,s_{t}\right)$,              ${\hat{\bar{t}}}_{m}\leftarrow C\left(b,s_{\bar{t}}\right)$3:Predict the sharp images on unlabeled data using *G* as:              ${\hat{s}}_{t}\leftarrow G\left(b,t^{\prime }\right)$,              ${\hat{s}}_{{\bar{t}}^{\prime }}\leftarrow G\left(b,{\bar{t}}^{\prime }\right)$4:Compute the weighting mask using $D_{d}$ and the sigmoid function as:              $\sigma_{t^{\prime }}\leftarrow \sigma \left(\left[D_{d}\left(b,G\left(b,t^{\prime }\right)\right)\right]\right)$,              $\sigma_{{\bar{t}}^{\prime }}\leftarrow \sigma \left(\left[D_{d}\left(b,G\left(b,{\bar{t}}^{\prime }\right)\right)\right]\right)$5:Compute $L_{C}^{\mathrm{CG}}$ by Equation (2) and $L_{C}^{L}$ by Equation (4)6:Compute the total loss $L_{C}$ by Equation (5)7:Update the parameters of *C* using the gradient of $L_{C}$ by Adam [36]

**Training of the generator** ***G*****.** To restore accurate pixel intensities, $L_{G}^{\mathrm{POC}}$ (Equation (1)) is utilized for optimizing *G*. To encourage *G* to restore a realistic sharp image according to arbitrary time codes, $L_{G}^{C}$ is defined using estimated time codes from *C*. Given the symmetric samples $\left\{b,t,\bar{t},s_{t},s_{\bar{t}}\right\}∼p_{d}$, the $\left({\hat{t}}_{m},{\hat{\bar{t}}}_{m}\right)$ can be obtained by ${\hat{t}}_{m}=C\left(b,G\left(b,t\right)\right)$ and ${\hat{\bar{t}}}_{m}=C\left(b,G\left(b,\bar{t}\right)\right)$. For randomly sampled time codes $\left\{t^{\prime },{\bar{t}}^{\prime }\right\}∼p_{t}$, we can also obtain ${\hat{t}}_{m}^{\prime }=C\left(b,G\left(b,t^{\prime }\right)\right)$ and ${\hat{\bar{t}}}_{m}^{\prime }=C\left(b,G\left(b,{\bar{t}}^{\prime }\right)\right)$. Then, our $L_{G}^{C}$ is formulated as follows:

(6) $$\begin{array}{ccc} \; & L_{G}^{C}=\; & \left\{\begin{array}{c} ∥{\hat{t}}_{m}-t_{m}{∥}_{2}+∥{\hat{\bar{t}}}_{m}-{\bar{t}}_{m}{∥}_{2}+∥{\hat{t}}_{m}^{\prime }-t_{m}^{\prime }{∥}_{2}+{∥{\hat{\bar{t}}}_{m}^{\prime }-{\bar{t}}_{m}^{\prime }∥}_{2}\;\mathrm{if}\;\Phi \left(s_{t},{\hat{s}}_{t}\right)<\Phi \left(s_{t},{\hat{s}}_{\bar{t}}\right)\\ ∥{\hat{t}}_{m}-{\bar{t}}_{m}{∥}_{2}+∥{\hat{\bar{t}}}_{m}-t_{m}{∥}_{2}+∥{\hat{t}}_{m}^{\prime }-{\bar{t}}_{m}^{\prime }{∥}_{2}+{∥{\hat{\bar{t}}}_{m}^{\prime }-t_{m}^{\prime }∥}_{2}\;\mathrm{otherwise}. \end{array}\right. \end{array}$$

To guarantee that the generated image is as realistic as the real data, the adversarial loss for *G* can be defined as follows:

(7) $$\begin{array}{ccc} L_{G}^{adv}\left(b,t\right) & =-[\log D_{e}\left(b,G\left(b,t\right)\right)+\; & \sum_{i,j}\log{\left[D_{d}\left(b,G\left(b,t\right)\right)\right]}_{\left(i,j\right)}]. \end{array}$$

Then, we define our adversarial loss $L_{G}^{D}$ using the symmetric pair of time codes $\left\{t,\bar{t}\right\}∼p_{d}$ and randomly sampled codes $\left\{t^{\prime },{\bar{t}}^{\prime }\right\}∼p_{t}$ as follows:

(8) $$\begin{array}{c} L_{G}^{D}=L_{G}^{adv}\left(b,t\right)+L_{G}^{adv}\left(b,\bar{t}\right)+L_{G}^{adv}\left(b,t^{\prime }\right)+L_{G}^{adv}\left(b,{\bar{t}}^{\prime }\right). \end{array}$$

Based on the above procedure, the entire objective of *G*, $L_{G}$, is formulated by the weighted sum of Equations (1), (6), and (8),

(9) $$\begin{array}{c} L_{G}=\lambda_{POC}L_{G}^{\mathrm{POC}}+\lambda_{C}L_{G}^{C}+\lambda_{D}L_{G}^{D}, \end{array}$$

where $\lambda_{POC}$, $\lambda_{C}$, and $\lambda_{D}$ are balancing weight parameters, which are empirically set to $\lambda_{POC}=1$, $\lambda_{C}=0.01$, and $\lambda_{D}=0.02$. Algorithm 4 shows the training scheme of *G*. As shown in line (2) of Algorithm 4, given the symmetric samples $\left\{b,t,s_{t},\bar{t},s_{\bar{t}}\right\}∼p_{d}$, the predicted deblurring results are obtained by ${\hat{s}}_{t}=G\left(b,t\right)$ and ${\hat{s}}_{\bar{t}}=G\left(b,\bar{t}\right)$. For unlabeled data $\left(t^{\prime },{\bar{t}}^{\prime }\right)∼p_{t}$, we can obtain the predicted deblurring results from *G* (line (3) of Algorithm 4). Then, predicted time code maps are obtained using *C*, as shown in lines (4) and (5) of Algorithm 4. Then, our proposed POC loss $L_{G}^{\mathrm{POC}}$ is obtained to restore more accurate pixel intensities on labeled data. $L_{G}^{C}$ allows our *G* to restore a realistic sharp image according to arbitrary time codes. The adversarial loss $L_{G}^{D}$ is computed to guarantee that the generated image is real for both labeled data and unlabeled data.
**Algorithm 4** Training of the generator *G*1:Sample a batch of labeled data $\left\{b,t,\bar{t},s_{t},s_{\bar{t}}\right\}∼p_{d}$ of size *n*,Sample a batch of unlabeled data $\left\{t^{\prime },{\bar{t}}^{\prime }\right\}∼p_{t}\left(t^{\prime }\right)$ of size *n*2:Predict the sharp images on labeled data using *G* as:              ${\hat{s}}_{t}\leftarrow G\left(b,t\right)$,              ${\hat{s}}_{\bar{t}}\leftarrow G\left(b,\bar{t}\right)$3:Predict the sharp images on unlabeled data using *G* as:              ${\hat{s}}_{t}^{\prime }\leftarrow G\left(b,t^{\prime }\right)$,              ${\hat{s}}_{{\bar{t}}^{\prime }}\leftarrow G\left(b,{\bar{t}}^{\prime }\right)$4:Predict the time code matrix on labeled data using *C* as:              ${\hat{t}}_{m}\leftarrow C\left(b,{\hat{s}}_{t}\right)$,              ${\hat{\bar{t}}}_{m}\leftarrow C\left(b,{\hat{s}}_{\bar{t}}\right)$5:Predict the time code matrix on unlabeled data using *C* as:              ${\hat{t}}_{m}^{\prime }\leftarrow C\left(b,{\hat{s}}_{t^{\prime }}\right)$,              ${\hat{{\bar{t}}^{\prime }}}_{m}\leftarrow C\left(b,{\hat{s}}_{{\bar{t}}^{\prime }}\right)$6:Compute $L_{G}^{\mathrm{POC}}$ by Equation (1), $L_{G}^{C}$ by Equation (6), and $L_{G}^{D}$ by Equation (8)7:Compute the total loss $L_{G}$ by Equation (9)8:Update the parameters of *G* using the gradient of $L_{G}$ by Adam [36]

## 4. Experiments

To evaluate and analyze our method, we performed various experiments. In the following subsections, the experimental setup is first explained, describing the implementation details, evaluation metrics, and datasets. Next, quantitative and qualitative comparisons are provided, demonstrating the superiority of the proposed method against previous competitive methods. Finally, an ablation study highlights the importance of the components of the proposed method.

### 4.1. Experimental Setup

**Implementation details.** The proposed ABDGAN was implemented using PyTorch 1.7.1 [37] and trained on NVIDIA TITAN-RTX GPUs. During training, the batch size *n* in Algorithm 1 was set to eight. For every iteration, the images were randomly cropped to a spatial size of 256×256×3, and a random horizontal flip was applied. The learning rates of *G*, *D*, and *C*, denoted as $\ell_{G},\ell_{D}$, and $\ell_{C}$, respectively, in Algorithm 1 were initialized identically as $1\times 10^{-4}$ and decayed exponentially by a factor of 0.99 for each epoch. Our ABDGAN was trained for 200 epochs. The Adam optimizer [36] was used with $\beta_{1}=0.9$ and $\beta_{2}=0.999$. In our ABDGAN, the time-conditional deblurring network *G* is built based on the NAFNet-GoPro-width32 base model [38], as detailed in Appendix B. Notably, we trained all the model parameters from scratch. For the discriminator *D*, we adopted the UNet discriminator [31] to provide per-pixel and per-image feedback to our *G* during training. The time-code predictor *C* is also based on the UNet architecture [35] used in BigGAN [39].

**Evaluation metrics.** For quantitative evaluation, we measured the peak signal-to-noise ratio (PSNR) and structural similarity index (SSIM) [40]. These are the most widely used metrics in image restoration tasks [28]. In addition, we used the learned perceptual image patch similarity (LPIPS) [41], which is commonly used to evaluate the perceptual quality. To investigate the computational costs of methods, we measured the number of model parameters, floating-point operations (FLOPs), and inference time. The FLOPS and inference time are measured using an input image with size of 256×256×3 following [19,38,42]. For a fair comparison, the inference time of the models were measured using a PC, which is equipped with a single NVIDIA TITAN RTX GPU and Intel(R) Xeon(R) Gold 5218 CPU. When comparing our model with existing blur decomposition methods [5,13], we calculated the FLOPs required to obtain a single sharp sequence from a single blurred image for each model. For example, since the official model of Jin et al. [5] is designed to obtain seven sharp frames from a blurred image, we measured the total FLOPs required to obtain seven sharp frames. Similarly, to compare with our model and Zhang et al. [13] that restores 15 sharp frames from a single blurred image, we measured the total FLOPs required to obtain 15 sharp frames. The inference time is calculated by averaging the time needed to restore 300 sharp video sequences from 300 input blurred images. When comparing our model with single image deblurring methods, the reported inference time represents the average across 300 images.

**Training and evaluation datasets.** We trained and evaluated our ABDGAN separately using GoPro [1] and B-Aist++[4] datasets. In this section, we denote each model trained with GoPro and B-Aist++ as “ABDGAN-GP” and “ABDGAN-BA”, respectively. While the original benchmark GoPro test set [1] contains blurred images synthesized by averaging more than 11 sharp frames per a single image, the models of Jin et al. [5] and Argaw et al. [6] were designed to restore seven frames from a single blurred image. For a fair comparison, we prepared the GoPro7 test set following Jin et al. [5]. Each blurred image is synthesized by averaging seven consecutive sharp frames provided by the official GoPro test set [1]. Meanwhile, the method of Zhang et al. [13] was proposed to extract 15 frames from a single blurred image. For a fair evaluation, we further prepared the GoPro15 test set by averaging 15 sharp frames of the official GoPro test videos. To evaluate the generalization performance of our proposed method, which was trained on the GoPro training set [1], we also evaluated the performance of our model on recent benchmark datasets such as REDS [3] and RealBlur [43]. While the GoPro training set and test set are synthesized using video captured with 240 fps, the REDS dataset [3] includes motion-blurred images synthesized using 120 fps video. Evaluating the model on the REDS dataset allows for a comprehensive assessment of its performance across various types of motion blur. This is significant despite the inherent differences in frame rates between the training data and the REDS dataset. The RealBlur dataset [43] provides the most prevalent scenarios for motion blur, i.e., low-light environments. Unlike the synthesis methodology used in GoPro and REDS, the RealBlur dataset [43] comprises pairs of the blurred and sharp images captured using their proposed dual camera system. Evaluating our model on this dataset allows us to assess its performance in handling common real-world motion blur conditions. Note that the REDS and RealBlur datasets are used only for the evaluation of our method trained on the GoPro training set.

A brief summary of the datasets is described as follows.

- **GoPro dataset**: We used the GoPro dataset [1], which is one of the most commonly used datasets for motion deblurring research. This dataset captures videos at 240 fps with a GoPro Hero camera and averages {7,9,11,13} frames for synthesizing blurred images. It provides a total of 3214 blurred images, of which 2103 are training images and 1111 are test images. Each image has a resolution of 1280×720. Following [5,6], we prepared the GoPro7 test set using the original GoPro test set. Unlike original GoPro test set that blurred images are synthesized by averaging more than 11, the GoPro7 test set is generated by averaging 7 sharp frames to create each corresponding blurred image. This GoPro7 test set comprises 1744 blurred images and the corresponding 12,208 sharp frames. Note that this test set is only used for evaluation purposes, as in [5,6]. Considering that [13] proposed to restore 15 frames from a single blurred image, the GoPro15 test set is prepared by averaging 15 sharp frames using the original GoPro test set. This test set consists of 811 blurry images and the corresponding 12,165 sharp frames.
- **B-Aist++ dataset**: Following [4], we utilized the B-Aist++ dataset [4], which consists of synthesized motion-blurred images using a human dancing video [44]. The dataset, respectively, contains 73 and 32 video clips for training and testing, and the spatial resolution of the images is 960×720.
- **REDS dataset**: The REDS dataset [3] provides realistic images of dynamic scene for image deblurring. It contains synthesized blurred images by merging sharp frames captured with 120 fps videos of 1280×720 resolution.
- **RealBlur dataset**: The RealBlur [43] dataset comprises blurred images captured in low-light static scenes. These blurred images simulate motion blur induced by camera shakes and captured in various low-light environments, including nighttime street scenes and indoor settings. The RealBlur test set consists of two subsets, RealBlur-J and RealBlur-R. The RealBlur-J test set contains JPEG images and the RealBlur-R test set contains images captured in the raw camera format. Each test set contains 980 image pairs of ground-truth and blurred images.

### 4.2. Quantitative Comparisons

**Blur decomposition.** To quantitatively evaluate the blur decomposition performance of the proposed method, we compared it with recent methods that generate multiple frames from a single blurred image. As the official codes of [6,11,12] are not released yet, we were only able to obtain the results of Jin et al. [5] and Zhang et al. [13] for testing on the benchmark GoPro dataset [1]. The results are shown in Table 3, where $F_{i},F_{m}$, and $F_{f}$ indicate the initial, middle, and last frame among the restored frames, respectively. “Avg.” denotes the average value of $F_{i},F_{m}$, and $F_{f}$. Following [4,6], the results of all methods are reported as the higher metric between the results of ground-truth order and reverse order due to the motion ambiguity [4,6]. The results demonstrate that our method outperforms other methods by a large margin in all metrics. Even though Zhang et al. [13] achieve the best result in terms of PSNR and SSIM for central frame prediction, their performance is highly biased towards the central frame. Jin et al. [5] show relatively more consistent performances between $F_{i},F_{m}$, and $F_{f}$ than those of Zhang et al. [13]. However, the method of Jin et al. [5] is limited to small motion due to their architecture and training procedure [11]. Hence, the performance of them often degrades when large and various degrees of blurred images are given, such as the GoPro benchmark test set. The quantitative comparisons on the GoPro7 test set and the GoPro15 test set are reported in Table 4 and Table 5, respectively. Since [6] do not provide the code and only report the PSNR and SSIM results in their paper, excluding LPIPS, we report only these metrics in Table 4. It is noteworthy that our method outperforms existing approaches in all metrics, including in restoring $F_{i},F_{m}$, and $F_{f}$ from a blurred image. This demonstrates that the sharp frames predicted by our ABDGAN are more consistent and of better quality than existing methods. Furthermore, the proposed method demonstrates superior performance in the GoPro7 test set compared to existing approaches specialized in predicting seven sharp frames from a single blurred image, such as Jin et al. [5] and Argaw et al. [6]. Moreover, when compared to Zhang et al. [13], which focuses on restoring 15 sharp frames, our method achieves superior results on the GoPro15 test set. These results highlight the superior generalization capabilities of our proposed method.

Table 6 shows the comparison of FLOPs, model parameters, and average inference time. Notably, our proposed ABDGAN-GP has 1.23× fewer FLOPs and runs 1.875× faster than Jin et al. [5]. Meanwhile, compared with Zhang et al. [13], our ABDGAN-GP performs with 1.10× larger FLOPs and is 2.31× slower. However, when considering the performance of deblurring accuracy in Table 3, our ABDGAN-GP demonstrates a more favorable trade-off between computational efficiency and deblurring accuracy compared to Jin et al. [5] and Zhang et al. [13].

For a fair comparison with the most recent blur decomposition method [4], we also trained and evaluated our ABDGAN with the B-Aist++ dataset [4]. Since existing methods [5,13] only released their test models (trained with GoPro dataset) without training code, we did not retrain them with the B-Aist++ dataset. The quantitative results are listed in Table 7. The results of Zhong et al. [4] were obtained using their motion predictor with a sampling number of five, which was reported as the best case in their paper. Even though the method of Zhong et al. [4] is effective in removing motion ambiguity by exploiting multiple motion guidances, the results show that our method can restore more accurate frames in terms of all metrics.

Most recent studies [5,6,13] in blur decomposition, including the proposed ABDGAN, utilize a synthetic motion blur dataset (i.e., GoPro dataset [1]) for training and evaluation. To compare the generalization abilities of these methods on real motion-blurred images, we conduct quantitative comparisons on RealBlur-R and RealBlur-J test sets [43]. The results are reported in Table 8. Since the official RealBlur-J and RealBlur-R test sets provide only a single ground-truth image for each blurred image in test sets, all metrics are computed based on the center frame prediction results. The results in Table 8 demonstrate that our method yields the best performance in all metrics. This indicates that the proposed ABDGAN can accurately restore sharp images from real motion-blurred scenes compared to existing methods [5,13].

**Center frame prediction.** Since most single image deblurring methods have been developed for predicting the center frame, we also conducted the quantitative comparison on central frames in Table 9. The results of our models were measured when the time code was set to t=0.5 during the test. The slight decrease in the performance of our ABDGAN compared to NafNet [38], which is a baseline architecture of our generator in terms of PSNR and SSIM, can be attributed to two factors. First, single image deblurring models, including NafNet, are trained specifically to predict only a single center frame from a blurred image. In contrast, our approach is a blur decomposition method that focuses on learning to extract an arbitrary sharp moment from a blurred image. Therefore, while the performance of the proposed method decreases in center prediction, our model has the ability to extract diverse sharp moments from a blurred image. Second, the integration of GANs within our method often tends to synthesize realistic but fake details [45,46]. Consequently, our model exhibits slightly lower PSNR and SSIM results compared to methods [17,18,19,38] that did not utilize GANs. However, it is noteworthy that our ABDGAN shows the best results in terms of PSNR and LPIPS among the GAN-based deblurring models, such as [1,5,12,22]. Due to the incorporation of our proposed temporal attention modules into NafNet, there is an increase in FLOPs, model parameters, and inference time compared to the original NafNet model. However, our proposed ABDGAN-GP remains competitive compared to other state-of-the-art deblurring models, as reported in Table 9. It achieves a balance between computational efficiency and deblurring performance.

### 4.3. Qualitative Comparisons

**Figure 3:**
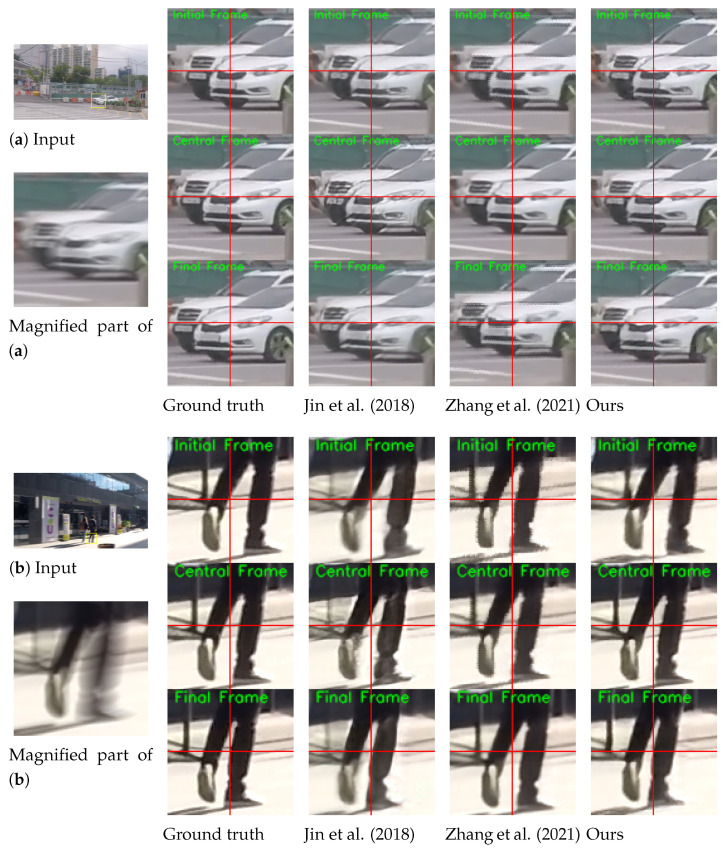
Qualitative comparison on official GoPro test set [1] compared with the single-to-video deblurring methods [5,13]. For clarity, we display the magnified parts of the output images. The initial, central, and final frames for each method are displayed. Horizontal and vertical lines are marked at the center coordinate of each image to provide clear observation of object movements between consecutive frames. *Please refer to the Supplementary Materials for comparisons on full video frames and the results of the proposed deblurring method*.

The visual results on the GoPro test set [1] are shown in Figure 3. The proposed ABDGAN outperforms all other methods [5,13] in extracting sharp and fine details. Moreover, the proposed method can generate plausible dynamic motion, whereas the results of other methods often appear to move globally. In Figure 3, when comparing the local movements of the two cars for input (a), the method of Jin et al. [5] produces overly smooth results. Although the results of Zhang et al. [13] contain images of visually pleasing quality, they fail to restore complex local motions, and all pixels tend to move globally. These observations can be found in most test results (e.g., see fine movements of the walking motion in the results of input (b)).

**Figure 4:**
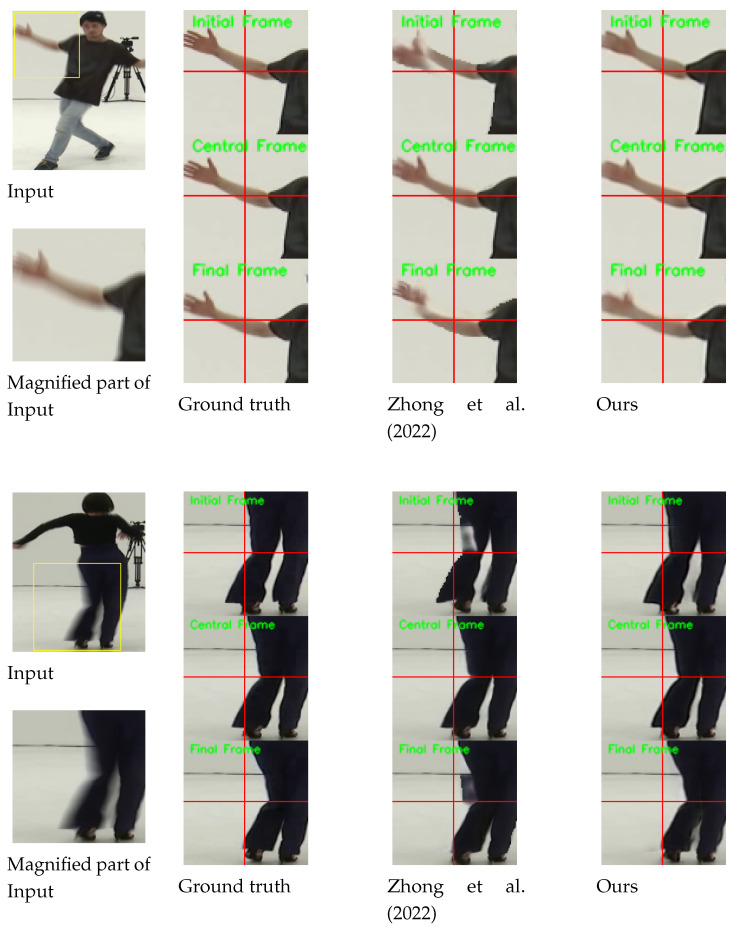
Qualitative comparison on the B-Aist++ test set [4]. For clarity, we display the magnified parts of the output images. Horizontal and vertical lines are marked at the center coordinate of each image to provide clear observation of object movements between consecutive frames. *Please refer to the Supplementary Materials for comparisons on full video frames and the results of the proposed deblurring method*.

Figure 4 shows the qualitative comparisons on the B-Aist++ test set [4]. While [4] fails to extract accurate motion of the dancers, our method can reconstruct more plausible motions (e.g., see the movements of the right hands in the first row and the movements of legs in the second and third rows).

**Figure 5:**
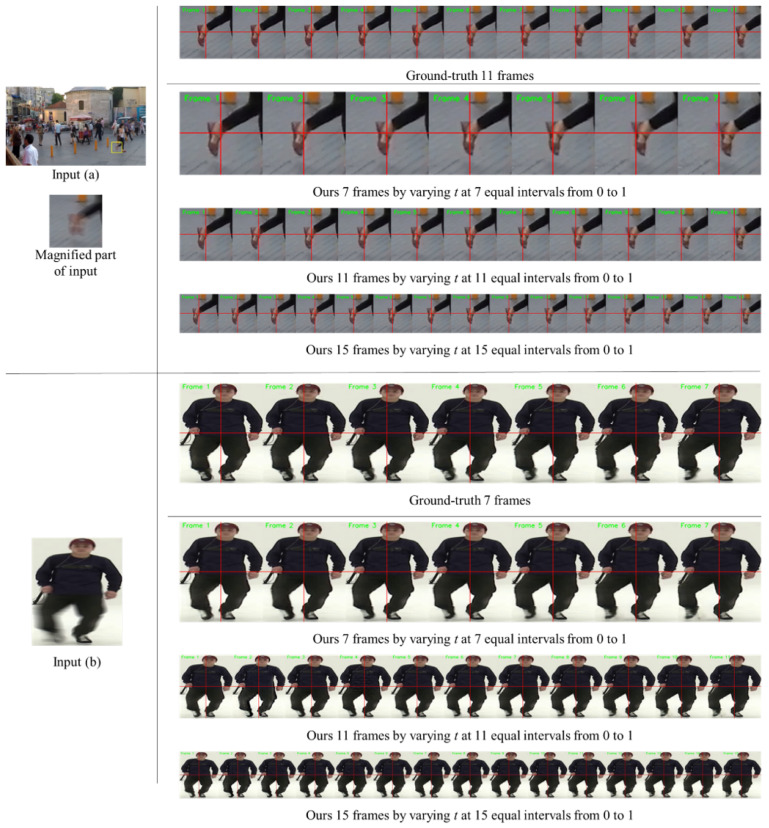
Several examples for the proposed ABDGAN on official GoPro test set [1] and B-Aist++ test set [4]. Note that our results are the outputs of the same network. With the adjustment of the input temporal code value *t*, our network restores any number of sharp moments from a given blurred image without architectural changes and retraining. To provide a clear observation for input (**a**), we display the magnified parts of the output images. Similarly, output images for input (**b**) are shown by varying time codes in our method. Horizontal and vertical lines are marked at the center coordinate of each image to provide clear observation of object movements between consecutive frames. *Please refer to the Supplementary Materials for more frames restored by the proposed deblurring method*.

Above all, the key aspect of our method is that our single model can accurately restore an arbitrary sharp moment from a blurred image. In our ABDGAN, these various outputs can be obtained by adjusting only the input time code *t* within [0,1]. Figure 5 shows the results of the proposed method of restoring flexible frame rates for the input image. Remarkably, our model consistently produces high-quality sharp frames with various number of frames, such as 7, 11, and 15 frames, without requiring any modification to the network architecture or retraining. In contrast, most of the existing blur decomposition methods [5,6,11,12] require a change of network architecture and retraining according to the number of outputs.

**Figure 6:**
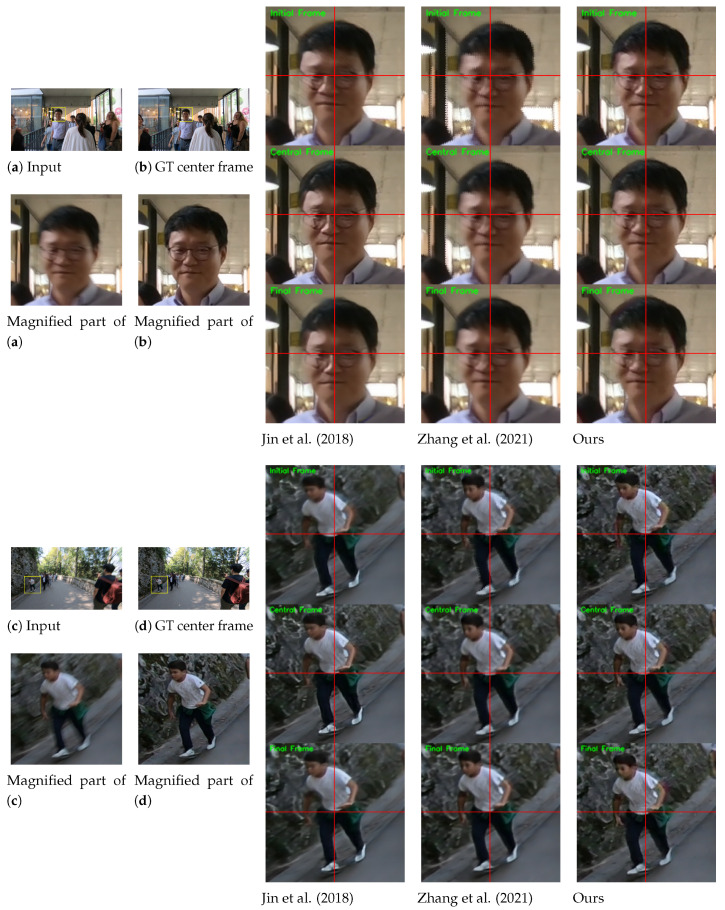
Qualitative comparison on REDS dataset [3] compared with the single-to-video deblurring methods [5,13]. For clarity, we display the magnified parts of the output images. Horizontal and vertical lines are marked at the center coordinate of each image to provide clear observation of object movements between consecutive frames. The initial, central, and final frames for each method are displayed. *Please refer to the Supplementary Materials for comparisons on full video frames and the results of the proposed deblurring method*.

The results on the REDS dataset [3] are provided in Figure 6. In this experiment, we use a validation set officially provided by [3]. The REDS validation set consists of pairs of blurred and central sharp frames. Hence, we only report these central frames as references for visual comparisons in this experiment. In Figure 6, it is observed that the proposed method restores the sharp frames with more realistic details than competing methods [5,13] (e.g., see the facial components of the glasses-wearing man in the results of input (a), and the pattern of the shirts in the results of input (c)).

**Figure 7:**
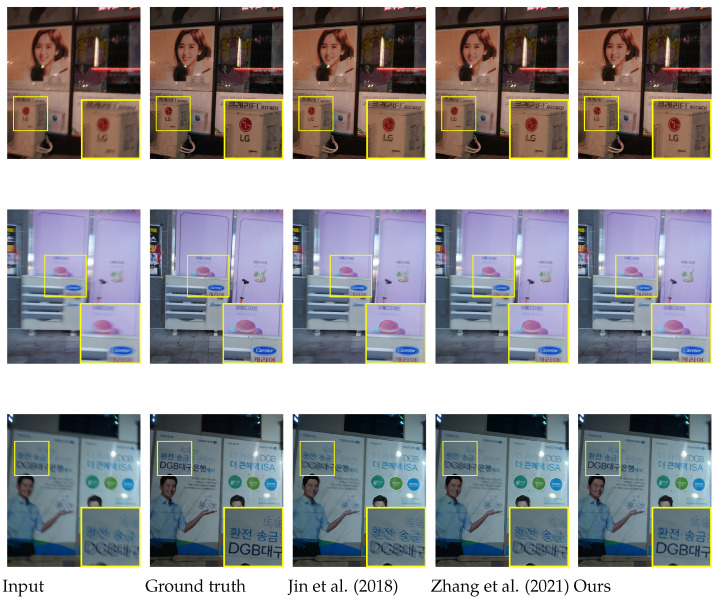
Qualitative comparison on official RealBlur test set [43] compared with the single-to-video deblurring methods [5,13].

Figure 7 shows the qualitative comparisons on the RealBlur test set [43]. The official RealBlur test set provides only a single sharp image per a single blurred image. Hence, we only compare the center frame results from Jin et al. [5], Zhang et al. [13] and our proposed ABDGAN-GP. The results demonstrate that our proposed method restores fine details in the sharp frames compared to the competing methods [5,13].

### 4.4. Ablation Study

We conducted ablation experiments to evaluate the impact of the components of our proposed approach. We trained four models, termed M1, M2, M3, and M4. All of these models shared the same architecture as *G*, but were trained with different loss functions. To solely compare the effects of the existing pairwise-order invariant loss ($L_{POI}$ in Equation (A1)) [5] and our proposed pairwise-order consistency loss ($L_{POC}$ in Equation (1)), we trained the M1 model using only $L_{POI}$ and the M2 model using only $L_{POC}$. Note that both M1 and M2 were trained in a supervised fashion without utilizing our *D* and *C*. Meanwhile, the M3 model and M4 model were trained by playing a min–max game consisting of our *G*, *D*, and *C*. The training time for both M1 and M2 is approximately 2 days each. For M3 and M4, the training time is approximately 3.5 days each.

As mentioned in Section 3.2, our CG loss allows our *C* to learn on trustworthy fake samples based on the critic guidance weight from *D*. Through this, our method mitigates the difficulty for *C* to predict the correct label, even if there is a distribution discrepancy between the real and fake images. To evaluate the effect of critic guidance weight from *D*, during training of the M3 model, we set both $\sigma_{t^{\prime }}$ and $\sigma_{{\bar{t}}^{\prime }}$ in Equation (2) to a value of 1 for all pixels. This indicates that our *C* was trained without critic guidance weight from *D*. In contrast, the M4 model was trained by taking advantage from critic guidance weight from *D*. Note that M4 is our proposed ABDGAN. In Table 10, we provide a brief summary of the configuration of the ablation models and present the quantitative comparisons.

**Figure 8:**
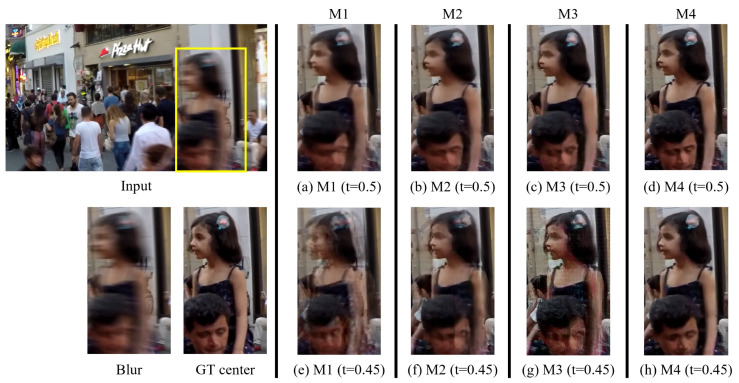
Ablation study of our method on the GoPro test dataset [1]. For clarity, we display the magnified parts of the output images.

The visual results in Figure 8 further highlight the effectiveness of the components within our proposed approach. Specifically, (a), (b), (c), and (d) in Figure 8 represent the outputs of M1, M2, M3, and M4, respectively. These outputs correspond to predicted images for the input time code t=0.5 that exists within the training data. Meanwhile, (e), (f), (g), and (h) in Figure 8 are the outputs of M1, M2, M3, and M4, respectively, when the input time code is set to 0.45. Notably, the time code 0.45 does not exist in the training data.

When comparing the results of M1 and M2 in Table 10, we can observe that our POC loss significantly improves the deblurring performance over the existing loss function of [5] for all metrics. The visual comparison of Figure 8a,b also shows that M2 produces higher-quality images than M1, which can be attributed to the use of our proposed POC loss. However, as shown in Figure 8e,f, both M1 and M2 models are unable to restore plausible images for unseen time codes during training. This indicates that models trained solely in a supervised fashion with a limited dataset tend to underperform. Specifically, these models struggle to generate sharp images when the time indices of the input blurred images are absent from the training set. Benefiting from using GANs, the quantitative performances of M3 and M4 are better than that of M2 in terms of LPIPS. This perceptual improvement is also observed in the visual comparison in Figure 8b–d. The visual comparisons in Figure 8g,h show that M4, which is trained with the CG loss, generates more realistic and sharper frames compared to M3. This indicates that the CG loss effectively guides the generator to produce more realistic frames by aligning the generated frame with the distribution of real sharp frames.

## 5. Limitations

**Figure 9:**
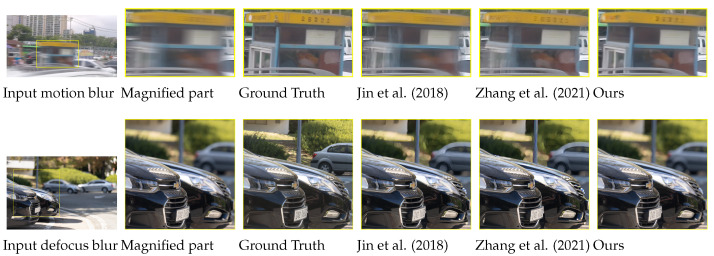
Failurecases of various methods [5,13], including the proposed ABDGAN.

Figure 9 shows the failure cases of our approach. The first row presents the results on a test image sampled from the benchmark GoPro test set [1]. In the second row, we show the outputs when the input blurred image is degraded by defocus blur. The input and the ground-truth images are sampled from the recent benchmark defocus blur test set proposed by Lee et al. [47]. Here, the results for central frames are shown for all models. Despite the significant advancements achieved by our proposed ABDGAN in motion-blur decomposition, we recognize two primary limitations. First, when the input image is severely degraded by substantial motion due to a large amount of local motion and camera shake, our method encounters challenges in accurately restoring a sharp frame, as shown in the first row in Figure 9. Second, since our method is specifically designed for restoring motion-blurred images, we did not account for various other types of blur (i.e., defocus blur) that commonly occur in real-world scenarios. As depicted in the second row in Figure 9, our method encounters limitations in such cases.

Based on these limitations, we believe that future research should focus on addressing severe motion blur. Additionally, improving the model’s robustness to accommodate various blur types, including defocus blur, remains a key focus for broader applicability.

## 6. Conclusions

In this paper, we proposed an ABDGAN, which is a novel approach for arbitrary time blur decomposition. By incorporating a TripleGAN-based framework, our ABDGAN learns to restore an arbitrary sharp moment latent in a given blurred image when the training data contain very few ground-truth images for continuous time code. We also proposed a POC loss that encourages our generator to restore more accurate pixel intensities. Moreover, we proposed a CG loss that ensures stability training by minimizing the distribution discrepancy between generated and real frames. Extensive experiments conducted on diverse benchmark motion blur datasets demonstrate the superior performance of our ABDGAN when compared to recent blur decomposition methods in terms of quantitative and qualitative evaluations.

The proposed ABDGAN outperforms the best competitor, enhancing PSNR, SSIM, and LPIPS on the GoPro test set by 16.67%, 9.16%, and 36.61%, respectively. On the B-Aist++ test set, our method provides improvements of 6.99% in PSNR, 2.38% in SSIM, and 17.05% in LPIPS over the best competitive method. In conclusion, the proposed ABDGAN restores arbitrary sharp moment from a single motion-blurred image with accurate, realistic, and pleasing quality. We believe that the proposed ABDGAN expands the application scope of image deblurring, which has traditionally focused on restoring a single image, to arbitrary time blur decomposition.

We anticipate that extending our ABDGAN to tackle a broader range of blur types, including defocus blur, will result in a more versatile and comprehensive deblurring solution. Future work will focus on enhancing the model’s capability to handle diverse blur scenarios, thereby improving its applicability and effectiveness in real-world situations.

## Figures and Tables

**Table 1:** Comparison of image deblurring and blur decomposition methods.

Category	Method	A Single Middle Frame Recovery	Fixed Multiple Frames Recovery	An Arbitrary Number of Multiple Frames Recovery
Image Deblurring	[1,14,15,16,17,18,19,20,21,22,23,24,25,26]	✓		
Blur Decomposition	[4,5,6,11,12,13,27]		✓	
	Our Approach			✓

**Table 2:** A summary of notations for the proposed ABDGAN.

Notation	Description
$b\in R^{H\times W\times 3}$	A motion-blurred image
t∈[0,1]	A time code that represents a specific moment in time within the exposure duration
$\bar{t}$	A symmetric time code to *t* with respect to central moment 0.5,
	which can be obtained by $\bar{t}=1-t$
$t^{\prime }$	A randomly sampled time code from the uniform distribution U[0,1].
$t_{m}\in R^{H\times W}$	A 2-dimensional matrix filled with *t*, as $t_{m(i,j)}=t$ for every pixel coordinate (i,j).
${\hat{t}}_{m}\in R^{H\times W}$	A 2-dimensional matrix predicted by label predictor *C* from *b* and *s*,
	which is defined by ${\hat{t}}_{m}=C\left(b,s\right)$, where *s* is either a real or predicted sharp image.
$s_{t}\in R^{H\times W\times 3}$	A sharp frame captured at time moment of t∈[0,1].
${\hat{s}}_{t}\in R^{H\times W\times 3}$	A predicted sharp frame using generator *G*, as ${\hat{s}}_{t}=G\left(b,t\right)$

**Table 3:** Quantitative comparison of video extraction performance on the GoPro test set [1]. $F_{i},F_{m}$, and $F_{f}$ indicate the initial, middle, and last frame among the restored frames, respectively. “Avg.” denotes the average value of $F_{i},F_{m}$, and $F_{f}$. The symbol ↑ in parentheses represents that the higher the value, the better. Similarly, the symbol ↓ indicates that the lower the value, the better. We highlight the best results and the second best results in **bold** and underline, respectively.

	Methods	$F_{i}$	$F_{m}$	$F_{f}$	Avg.
	Jin et al. [5]	24.04	26.98	23.12	24.71
PSNR (↑)	Zhang et al. [13]	18.78	**31.05**	18.75	22.87
	ABDGAN-GP	**28.16**	30.16	**28.18**	**28.83**
	Jin et al. [5]	0.833	0.881	0.810	0.841
SSIM (↑)	Zhang et al. [13]	0.688	**0.949**	0.672	0.770
	ABDGAN-GP	**0.911**	0.934	**0.910**	**0.918**
	Jin et al. [5]	0.308	0.255	0.323	0.295
LPIPS (↓)	Zhang et al. [13]	0.428	0.192	0.442	0.354
	ABDGAN-GP	**0.205**	**0.149**	**0.207**	**0.187**

**Table 4:** Quantitative comparison of video extraction performance on the GoPro7 test set. $F_{i},F_{m}$, and $F_{f}$ indicate the initial, middle, and last frame among the restored frames, respectively. “Avg.” denotes the average value of $F_{i},F_{m}$, and $F_{f}$. The symbol ↑ in parentheses represents that the higher the value, the better. Similarly, the symbol ↓ indicates that the lower the value, the better. We highlight the best results and the second best results in **bold** and underline, respectively.

	Methods	$F_{i}$	$F_{m}$	$F_{f}$	Avg.
	Jin et al. [5]	23.71	29.47	23.68	24.71
PSNR (↑)	Argaw et al. [6]	27.36	31.99	27.41	28.92
	ABDGAN-GP	**28.29**	**32.38**	**28.31**	**29.66**
	Jin et al. [5]	0.660	0.846	.659	0.722
SSIM (↑)	Argaw et al. [6]	0.794	0.885	0.793	0.824
	ABDGAN-GP	**0.873**	**0.942**	**0.882**	**0.899**
	Jin et al. [5]	0.301	0.190	0.307	0.266
LPIPS (↓)	Argaw et al. [6]	N/A	N/A	N/A	N/A
	ABDGAN-GP	**0.191**	**0.145**	**0.201**	**0.179**

**Table 5:** Quantitative comparison of video extraction performance on the GoPro15 test set. $F_{i},F_{m}$, and $F_{f}$ indicate the initial, middle, and last frame among the restored frames, respectively. “Avg.” denotes the average value of $F_{i},F_{m}$, and $F_{f}$. The symbol ↑ in parentheses represents that the higher the value, the better. Similarly, the symbol ↓ indicates that the lower the value, the better. The best results are highlighted in **bold**, respectively.

	Methods	$F_{i}$	$F_{m}$	$F_{f}$	Avg.
PSNR (↑)	Zhang et al. [13]	17.81	28.84	17.52	21.39
	ABDGAN-GP	**21.08**	**28.88**	**21.62**	**23.86**
SSIM (↑)	Zhang et al. [13]	0.639	0.805	0.638	0.694
	ABDGAN-GP	**0.735**	**0.874**	**0.743**	**0.781**
LPIPS (↓)	Zhang et al. [13]	0.432	0.274	0.443	0.383
	ABDGAN-GP	**0.351**	**0.217**	**0.346**	**0.305**

**Table 6:** Quantitative comparison on FLOPs, inference time, and model parameters.

Methods	FLOPs(G)		Inference Time (s)		Params. (M)
	**7 Frames**	**15 Frames**	**7 Frames**	**15 Frames**	
Jin et al. [5]	426.0	N/A	0.45	N/A	18.2
Zhang et al. [13]	N/A	673.7	N/A	0.22	26.3
ABDGAN-GP	345.8	741.1	0.24	0.51	25.9

**Table 7:** Quantitative comparison of video extraction performance on the B-Aist++ test set [4]. The symbol ↑ in parentheses represents that the higher the value, the better. Similarly, the symbol ↓ indicates that the lower the value, the better. We highlight the best results in **bold**, respectively.

	Methods	$F_{i}$	$F_{m}$	$F_{f}$	Avg.
PSNR (↑)	Zhong et al. [4]	19.42	30.66	19.46	23.18
	ABDGAN-BA	**21.50**	**31.38**	**21.53**	**24.80**
SSIM (↑)	Zhong et al. [4]	0.846	0.954	0.846	0.882
	ABDGAN-BA	**0.877**	**0.957**	**0.874**	**0.903**
LPIPS (↓)	Zhong et al. [4]	0.157	0.076	0.155	0.129
	ABDGAN-BA	**0.126**	**0.068**	**0.126**	**0.107**

**Table 8:** Quantitative comparison of the center frame prediction on the RealBlur test dataset [43]. The symbol ↑ in parentheses represents that the higher the value, the better. Similarly, the symbol ↓ indicates that the lower the value, the better. We highlight the best and the second best results in **bold** and underline, respectively.

Methods	RealBlur-J			RealBlur-R		
	**PSNR(**↑**)**	**SSIM(**↑**)**	**LPIPS(**↓**)**	**PSNR(**↑**)**	**SSIM(**↑**)**	**LPIPS(**↓**)**
Jin et al. [5]	26.30	0.803	0.283	32.15	0.908	0.236
Zhang et al. [13]	26.37	0.802	0.266	31.81	0.893	0.264
ABDGAN-GP	**26.77**	**0.818**	**0.234**	**33.23**	**0.926**	**0.184**

**Table 9:** Quantitative comparison of the center frame prediction on the GoPro test dataset [1]. The methods (1st to 6th row) are the single image deblurring models that are trained to restore only center frames. When computing the FLOPs, the image size is set as 3×256×256. The symbol ↑ in parentheses represents that the higher the value, the better. Similarly, the symbol ↓ indicates that the lower the value, the better. We highlight the best and the second best results in **bold** and underline, respectively.

Method	PSNR (↑)	SSIM (↑)	LPIPS (↓)	FLOPs (G)	Params. (M)	Inference Time (s)
DeepDeblur [1]	29.23	0.916	0.231	1760.04	11.7	4.36
SRN [17]	30.26	0.934	0.226	1434.82	6.8	1.88
DMPHN [18]	31.20	0.945	0.207	195.44	21.7	0.427
DBGAN [22]	30.43	0.937	0.191	1519.7	11.6	0.097
MIMO-UNet [19]	32.45	0.957	0.185	150.68	16.1	0.017
NafNet_32_ [38]	**32.87**	**0.960**	0.176	32.20	17.1	0.024
ABDGAN-GP	30.16	0.934	**0.149**	50.20	25.9	0.033

**Table 10:** Effects of the components of our ABDGAN on the GoPro test set [1]. The reported metrics are averaged results measured with all predicted frames and GT frames (12,221 images). The symbol ↑ in parentheses represents that the higher the value, the better. Similarly, the symbol ↓ indicates that the lower the value, the better.

ABDGAN-GP	M1	M2	M3	M4
Pairwise-order invariant loss [5]	✓	×	×	×
Pairwise-order consistency loss	×	✓	✓	✓
without critic guidance weight from *D*	×	×	✓	×
with critic guidance weight from *D*	×	×	×	✓
PSNR (↑)	27.17	28.29	28.28	28.24
SSIM (↑)	0.879	0.911	0.899	0.910
LPIPS (↓)	0.207	0.195	0.184	0.180

**Figure A1:**
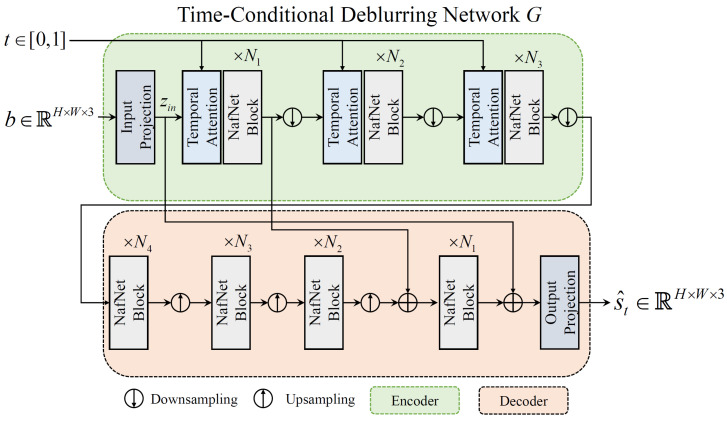
The proposed time-conditional deblurring network.

**Figure A2:**
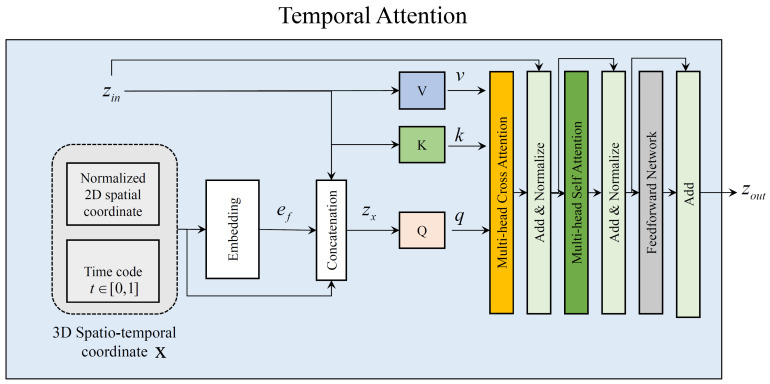
The proposed temporal attention module.

## Data Availability

Data are contained within the article.

## Supplementary Materials

The following supporting information can be downloaded at: https://www.mdpi.com/article/10.3390/s24154801/s1.

## Appendix A. Drawback of POI Loss

In the following, we describe the limitations of the existing POI loss [5]. To avoid any confusion, unless stated otherwise, we use the notation consistent with the main paper.

Let $\left\{b,t,s_{t},\bar{t},s_{\bar{t}}\right\}∼p_{d}$ denote the sampled set from the dataset, where $\bar{t}=1-t$, and $\left(s_{t},s_{\bar{t}}\right)$ represents a pair of symmetric ground-truth frames for the central frame $s_{t=0.5}$. We denote ${\hat{s}}_{t}$ and ${\hat{s}}_{\bar{t}}$ as the frames predicted by *G* as ${\hat{s}}_{t}=G\left(b,t\right)$ and ${\hat{s}}_{\bar{t}}=G\left(b,\bar{t}\right)$, respectively. Then, existing POI loss $L_{POI}$ [5] is defined as follows:

(A1) $$\begin{array}{ccc} \; & L_{POI}\; & =\underset{L_{\mathrm{sum}}}{\underbrace{|∥{\hat{s}}_{t}+{\hat{s}}_{\bar{t}}{∥}_{1}-{∥s_{t}+s_{\bar{t}}∥}_{1}|}}+\underset{L_{\mathrm{diff}}}{\underbrace{|∥{\hat{s}}_{t}-{\hat{s}}_{\bar{t}}{∥}_{1}-{∥s_{t}-s_{\bar{t}}∥}_{1}|}}. \end{array}$$

As shown in Equation (A1), $L_{POI}$ comprises two components, $L_{\mathrm{sum}}$ and $L_{\mathrm{diff}}$. $L_{\mathrm{sum}}$ is defined as the absolute difference between the $\ell_{1}$ norms of the sum of predicted time-symmetric frames (${\hat{s}}_{t}$ and ${\hat{s}}_{\bar{t}}$) and their corresponding GT time-symmetric frames ($s_{t}$ and $s_{\bar{t}}$). On the other hand, $L_{\mathrm{diff}}$ quantifies the absolute difference between the $\ell_{1}$ norms of the difference between the predicted frames and the ground-truth frames. Alternatively, $L_{\mathrm{sum}}$ can be expressed in a pixel-wise manner as follows:

(A2) $$\begin{array}{cc} L_{\mathrm{sum}} & =|\sum_{i,j}\left|{\hat{s}}_{t(i,j)}+{\hat{s}}_{\bar{t}\left(i,j\right)}\right|-\sum_{i,j}\left|s_{t(i,j)}+s_{\bar{t}\left(i,j\right)}\right||\\ \; & =|\sum_{i,j}\left(({\hat{s}}_{t(i,j)}+{\hat{s}}_{\bar{t}\left(i,j\right)}\right)-\left(s_{t(i,j)}+s_{\bar{t}\left(i,j\right)}\right))|, \end{array}$$

where (i,j) indicates the pixel coordinate. Since $L_{\mathrm{sum}}$ focuses on the sum of the symmetric GT frames, it can potentially lead to a behavior where the predicted pixel values of the symmetric frames are similar to the sum of their ground-truth counterparts. Consequently, $L_{\mathrm{sum}}$ still has ambiguity in predicting the temporal order of frames. For instance, regardless of whether the temporal order of predicted frames is correct or reversed, their sum might still match the sum of the ground-truth frames. Therefore, the pixel-level temporal order consistency is not assured.

Moreover, we argue that $L_{\mathrm{diff}}$ may result in suboptimal solutions during network training. If we rephrase the $L_{\mathrm{diff}}$ in a pixel-wise manner, it can be expressed as follows:

(A3a) $$\begin{array}{cc} L_{\mathrm{diff}} & =|\sum_{i,j}\left|{\hat{s}}_{t(i,j)}-{\hat{s}}_{\bar{t}\left(i,j\right)}\right|-\sum_{i,j}\left|s_{t(i,j)}-s_{\bar{t}\left(i,j\right)}\right|| \end{array}$$

(A3b) $$\begin{array}{cc} \; & =|\sum_{i,j}(\underset{\left(a\right)}{\underbrace{\left|{\hat{s}}_{t(i,j)}-{\hat{s}}_{\bar{t}\left(i,j\right)}\right|}}-\underset{\left(b\right)}{\underbrace{\left|s_{t(i,j)}-s_{\bar{t}\left(i,j\right)}\right|}})|, \end{array}$$

where (i,j) represents the pixel coordinate. Depending on the signs of term (a) and (b) in Equation (A3b), this can branch into four different cases, as illustrated in Table A1. It is noteworthy that ${\hat{s}}_{t(i,j)}$ can be matched by either $s_{t(i,j)}$ (1st and 2nd rows in Table A1) or $s_{\bar{t}\left(i,j\right)}$ (3rd and 4th rows in Table A1). Simultaneously, ${\hat{s}}_{\bar{t}\left(i,j\right)}$ can correspond to either $s_{\bar{t}\left(i,j\right)}$ (1st and 2nd rows in Table A1) or $s_{t(i,j)}$ (3rd and 4 rows in Table A1). This condition implies that each pixel in a single predicted image can be influenced by pixels from two different time-symmetric GT frames, rather than solely by pixels from a single GT image. This may impede the network’s ability to consistently learn the temporal order of the GT frames.

**Table A1:** Four cases in Equation (A3b).

Signs of (a) & (b)	$\left|{\hat{s}}_{t(i,j)}-{\hat{s}}_{\bar{t}\left(i,j\right)}\right|-\left|s_{t(i,j)}-s_{\bar{t}\left(i,j\right)}\right|$ in Equation (A3b)
(a)≥0&(b)≥0	${\hat{s}}_{t(i,j)}-{\hat{s}}_{\bar{t}\left(i,j\right)}-s_{t(i,j)}+s_{\bar{t}\left(i,j\right)}=\left({\hat{s}}_{t(i,j)}-s_{t(i,j)}\right)-\left({\hat{s}}_{\bar{t}\left(i,j\right)}-s_{\bar{t}\left(i,j\right)}\right)$
(a)<0&(b)<0	$-{\hat{s}}_{t(i,j)}+{\hat{s}}_{\bar{t}\left(i,j\right)}+s_{t(i,j)}-s_{\bar{t}\left(i,j\right)}=-\left({\hat{s}}_{t(i,j)}-s_{t(i,j)}\right)+\left({\hat{s}}_{\bar{t}\left(i,j\right)}-s_{\bar{t}\left(i,j\right)}\right)$
(a)≥0&(b)<0	${\hat{s}}_{t(i,j)}-{\hat{s}}_{\bar{t}\left(i,j\right)}+s_{t(i,j)}-s_{\bar{t}\left(i,j\right)}=\left({\hat{s}}_{t(i,j)}-s_{\bar{t}\left(i,j\right)}\right)-\left({\hat{s}}_{\bar{t}\left(i,j\right)}-s_{t(i,j)}\right)$
(a)<0&(b)≥0	${\hat{s}}_{t(i,j)}-{\hat{s}}_{\bar{t}\left(i,j\right)}+s_{t(i,j)}-s_{\bar{t}\left(i,j\right)}=-\left({\hat{s}}_{t(i,j)}-s_{\bar{t}\left(i,j\right)}\right)+\left({\hat{s}}_{\bar{t}\left(i,j\right)}-s_{t(i,j)}\right)$

## Appendix B. Time-Conditional Deblurring Network

The architectural overview of the time-conditional deblurring network *G* is illustrated in Figure A1. Since our baseline architecture, NafNet [38], is designed as an UNet-like structure [35], our *G* consists of an encoder and a decoder. In Figure A1, “NafNet Block” indicates the unit block proposed in Chen et al. [38]. Inspired by [48], we design the temporal attention module to find the most salient sharp image corresponding to the specific spatiotemporal coordinates within the entangled sharp images in a blurred image. This module allows the deblurring network to learn not only the complex motion of the objects but also to selectively focus on different sharp representations in a blurred image according to the input time code. This level of specificity could be difficult to model using 2D or 3D convolutions [49]. The temporal attention module can be flexibly plugged into many single deblurring networks designed for accurate and effective restoration.

In more detail, given a blurred image $b\in R^{H\times W\times 3}$, the proposed *G* applies a 3×3 convolution layer to extract feature maps $z_{in}\in R^{H\times W\times C}$, which is denoted as the “Input Projection” layer in Figure A1. Then, extracted feature maps $z_{in}$ and input temporal code *t* are fed into the proposed temporal attention module, which is termed as “Temporal Attention” in Figure A2. The proposed temporal attention module consists of a sequence of layers, where each layer includes multihead cross attention (MCA), multihead self attention (MSA), and a feedforward network (FFN). Then, our module can be expressed as follows:

(A4) $$\begin{array}{cc} \; & e_{f}=\mathrm{Embedding}\left(x\right),\\ \; & z_{x}=\mathrm{Concatenation}\left[x,e_{f},z_{in}\right],\\ \; & y_{0}=z_{in}+\mathrm{MCA}\left(z_{x},z_{in}\right),\\ \; & y_{0}^{\mathrm{LN}}=\mathrm{LN}\left(y_{0}\right)\\ \; & y_{1}=y_{0}+\mathrm{MSA}\left(y_{0}^{\mathrm{LN}}\right),\\ \; & y_{1}^{\mathrm{LN}}=\mathrm{LN}\left(y_{1}\right)\\ \; & z_{out}=y_{1}+\mathrm{FFN}\left(y_{1}^{\mathrm{LN}}\right), \end{array}$$

where x is the 3D spatiotemporal coordinates and LN is the layer normalization [50]. Here, $x_{\left(i,j,t\right)}=\left[\frac{2i}{H-1}-1,\frac{2j}{W-1}-1,t\right],i\in \left[0,H-1\right],j\in \left[0,W-1\right],t\in \left[0,1\right]$. Following [51], we normalize the spatial coordinates to the range of [−1,1] in $x\in R^{H\times W\times 3}$. Inspired by [52], which utilized both a Fourier embedding feature map and spatial coordinate information for producing high-frequency components of the images with fewer artifacts, we apply the Fourier embedding [52] to x. Specifically, the Fourier embedded feature map $e_{f}\in R^{H\times W\times C}$ is obtained by applying 1×1 convolution layer followed by sine activation function to x, which is defined by $e_{f}=\sin\left[1\times 1\mathrm{conv}\left(x\right)\right]$. In Equation (A4), we denote this Fourier embedding process as “Embedding(·)” for simplicity. Then, we obtain $z_{x}$ by concatenation of spatiotemporal coordinates x, Fourier features $e_{f}$, and image feature $z_{in}$, which is defined as follows: $z_{x}=\mathrm{Concatenation}\left[x,e_{f},z_{in}\right]$. The $z_{x}$ is projected to query feature map, and $z_{in}$ is projected to key and value feature maps for cross-attention computation [48].

Inspired by [53], who proposed an efficient attention layer by reducing memory costs for image restoration, 3×3 depth-wise convolution and 1×1 point-wise convolution are used in each *Q*, *K*, and *V* layer. Concretely, the cross-attention MCA can be formulated as follows:

(A5) q=WqdWqpzx,k=WkdWkpzin,v=WvdWvpzin,B=softmax(qkTqkTc/mc/m),MCA(zx,zin)=Bv,

where $W_{\left(\cdot \right)}^{d}$ and $W_{\left(\cdot \right)}^{p}$ represent the learnable weights of 3×3 depth-wise convolution and 1×1 point-wise convolution, respectively. *c* and *m* are the embedding dimension and the number of heads. B represents the attention map obtained by dot production between *q* and *k*, followed by the softmax function as in [48]. The self attention MSA follows similar process in Equation A5, but the input variable for the Q,K and *V* layer is the same as $y_{0}^{LN}$. The FFN includes two layers of multilayer perceptrons. We apply the GELU activation function [54] between the two layers of the FFN and use layer normalization [50].

In this work, following [38], the numbers of the NafNet blocks in the encoder and decoder, which are termed N1,N2,N3, and N4 in Figure A1, are set to 1,1,1, and 28, respectively. For temporal attention modules in our *G*, the number of heads *m* in Equation A5 are all set to 1. We set the embedding dimension *c* in Equation A5 as equal to the dimension of the input $z_{in}$ for the temporal attention module.
